# A comparison of tau aggregation and seeding competency of anti‐tau antibodies under clinical trials and their target epitopes itself

**DOI:** 10.1002/alz.088690

**Published:** 2025-01-09

**Authors:** Min‐Seok Kim, Ha‐Lim Song, Seung‐Yong Yoon

**Affiliations:** ^1^ ADEL Institute of Science & Technology (AIST), ADEL, Inc., Seoul Korea, Republic of (South); ^2^ Asan Medical Center, University of Ulsan College of Medicine, Seoul Korea, Republic of (South)

## Abstract

**Background:**

Abnormal aggregation and accumulation of tau is a hallmark of tauopathy including Alzheimer’s disease. Effective targeting of tau for therapeutic purposes requires a clear understanding of its epitope landscape with identification of a key pathogenic tau species. Despite numerous proposed and tested tau epitopes, ranging from the N‐terminus to the microtubule‐binding region and C‐terminus, the most effective target remains elusive.

**Method:**

We compared the impact of tau aggregation and seeding using various peptides representing epitopes of anti‐tau antibodies in clinical trials or fragments of the MTBR found in the cerebrospinal fluid of tauopathies. We also evaluated the effects of several tau antibodies on the inhibition of tau aggregation and seeding using recombinant tau protein or AD brain extracts.

**Result:**

Peptide containing acetylated lysine‐280 was most potent on tau seeding measured by tau‐FRET and on inducing tau aggregation evaluated by thioflavin T compared to other peptides. Further, inhibition of tau aggregation and seeding was most prominent in anti‐acetylated lysine‐280 antibody‐treated cells.

**Conclusion:**

Our results demonstrate that acetylated lysine‐280 is the most potent inducer of tau aggregation and seeding. These findings offer valuable insights into the design of tau‐targeted therapeutics and highlight acetylated lysine‐280 as a promising target for the treatment of tauopathies.

